# The Gut's Little Brain in Control of Intestinal Immunity

**DOI:** 10.1155/2013/630159

**Published:** 2013-04-04

**Authors:** Wouter J. de Jonge

**Affiliations:** Tytgat Institute for Gastrointestinal and Liver Research, Department of Gastroenterology, Academic Medical Centre, Meibergdreef 69, 1105 NH Amsterdam, The Netherlands

## Abstract

The gut immune system shares many mediators and receptors with the autonomic nervous system. Good examples thereof are the parasympathetic (vagal) and sympathetic neurotransmitters, for which many immune cell types in a gut context express receptors or enzymes required for their synthesis. For some of these the relevance for immune regulation has been recently defined. Earlier and more recent studies in neuroscience and immunology have indicated the anatomical and cellular basis for bidirectional interactions between the nervous and immune systems. Sympathetic immune modulation is well described earlier, and in the last decade the parasympathetic vagal nerve has been put forward as an integral part of an immune regulation network via its release of Ach, a system coined “the cholinergic anti-inflammatory reflex.” A prototypical example is the inflammatory reflex, comprised of an afferent arm that senses inflammation and an efferent arm: the cholinergic anti-inflammatory pathway, that inhibits innate immune responses. In this paper, the current understanding of how innate mucosal immunity can be influenced by the neuronal system is summarized, and cell types and receptors involved in this interaction will be highlighted. Focus will be given on the direct neuronal regulatory mechanisms, as well as current advances regarding the role of microbes in modulating communication in the gut-brain axis.

## 1. Introduction

### 1.1. Innervation and Immunity in the Gastrointestinal Tract

It is increasingly acknowledged that the neuroendocrine and, the sympathetic and parasympathetic arms of the autonomic nervous system and the enteric nervous system are the key pathways through which the gut and the brain communicate. The principle is established that the immune system can no longer be regarded as an autonomous functioning entity but clearly receives regulatory input from neuronal systems. The “cholinergic inflammatory neuronal reflex” is one example of how action potentials originating in neurons influence immunity. The lymphoid organs of the immune system are innervated by cholinergic, catecholaminergic, and peptidergic, and other neurons, and many neurotransmitters and receptors are shared between the immune system and the nervous system, substantiating claims of a strong regulatory component of the nervous system in immune responses. A good example thereof is the discovery of acetylcholine producing memory T-cells in the circulation [1, 2] and spleen [3]. It is likely that such cells can interact with other antigen presenting cells or feed back on nerve terminals in primary or secondary lymphoid organs. This evidence of neuroimmune intercommunication in gut immunity are discussed in the subsequent sections. This paper highlights the role of selected neuromediators in regulating immunity, with a particular focus on myeloid immune cells. Neurotransmitters clearly have a marked and often diverse influence on macrophages and dendritic cells depending on the local microenvironment, the differentiation, and activation states of the cell. Notwithstanding the complex interplay between neuromediators and immune cells, the careful evaluation of receptor agonists and antagonists in inflammatory disease models may provide new therapeutic avenues for the treatment of human pathologies.

The central nervous system (CNS) communicates with the intestine through what is known as the brain-gut axis, comprising of the hypothalamic-pituitary-adrenal (HPA) axis and, in a gut context, the autonomic enteric nervous system (ENS) within the intestinal wall. Both vagal and spinal sensory neurons terminate at various points within the gut wall including the muscularis and the mucosal epithelium and play an important role in the transfer of information from the CNS to the ENS, and vice versa [4, 5]. Vagal fibers innervate the myenteric plexus in the colon and the ileum, whilst cholinergic fibers found in the gut mucosa are most likely to be a part of the ENS that forms a dense multidirectional network composed of efferent, afferent interneurons and the enteric glial cells. These neural projections obviously play an important regulating role in gastrointestinal motility, secretion, barrier function, and so forth, but seem an equally important regulatory arm for the inflammatory response at the mucosa (see for a recent review [6] amongst others). 

### 1.2. The Anatomy of Parasympathetic and Sympathetic Pathways

The ANS is largely autonomous (independent) in that its activities are not under direct conscious control. The sympathetic nervous system is typically associated with the physiological “fight or flight” response, such that it is involved in the regulation of cardiovascular and respiratory function, especially during times of critical need. In addition, the sympathetic nervous system regulates gastrointestinal tract smooth muscle contraction/relaxation, gastric secretions, and other autonomic functions. The ANS consists of three components: the sympathetic (noradrenergic) and parasympathetic (cholinergic) systems, which originate in the CNS (with cell bodies in the brainstem and spinal cord), and the enteric system, which lies within the wall of the gastrointestinal tract. The most extensive and physiologically most diverse component is the SNS, which sends axons to all parts of the body. The enteric system, which contains a similar number of neurons as the spinal cord [7], regulates intestinal functions; this system is modulated by projections from the sympathetic and the parasympathetic systems. Anatomical evidence is available to show that lymphoid follicles are innervated by noradrenergic fibers [8]. Parasympathetic (mainly vagal) reflexes coordinate gastric and intestinal digestive functions and motility. These functions are controlled through functionally distinct pathways, coined neurocircuitries, connecting the intestine with the brain stem nuclei, that is, the nucleus of the solitary tract (NTS) and the dorsal motor nucleus of the vagus (DMV). Mechanical (i.e., contraction, distention) and chemical (i.e., nutrients) signals are transmitted to the NTS via the vagal ascending fibers. After integration of the incoming information and through neuronal communication between the NTS and the DMV, vagal efferent output is triggered to monitor and regulate gastrointestinal functions such as secretion, absorption, and motility [9, 10]. However, it should be noted that parasympathetic and sympathetic systems closely interact, as is, for instance, illustrated by the observation that the vagus nerve can inhibit the sympathetic thoracic nerves to release pancreatic norepinephrine [11], or enhance splenic norepinephrine, and many other examples thereof exist. 

### 1.3. Enteric Innervation of the GI Tract (ENS)

The central nervous system (CNS) communicates with the GI tract in a bidirectional fashion largely through the enteric nervous system (ENS). The autonomic ENS comprises parasympathetic and sympathetic systems that can operate without the participation of the CNS, although the ENS interacts directly with the CNS through (para)sympathetic nerves (i.e., spinal/splanchnic reflexes). The neural ganglia within the ENS are organized in several plexuses throughout the intestinal wall: the myenteric (or Auerbach's plexus, between circular and longitudinal muscle layer) and submucosal (or Meissner's plexus, in the submucosa) plexuses. The mucosal layer also contains nerve networks known as the mucosal plexus, which contains nerve endings that are potentially in contact with mucosal Antigen Presenting Cells (APCs), although the exact nature of such associations is to be determined. The ENS contains sensory neurons, interneurons, and motor neurons, which primarily control peristalsis, local changes in blood flow, and secretion of water and electrolytes. Important components of the ENS are the supportive enteric glial cells (EGCs), which form a large and widespread network at all levels of the gastrointestinal tract and serve as intermediaries in the enteric neurotransmission and information processing. More than 30 different efferent and afferent neurotransmitters exist in the ENS, with most neurons expressing multiple transmitters. Like neurons of the central nervous system, ENS neurons secrete acetylcholine (ACh) and large number of other neurotransmitters and neuropeptides including norepinephrine (NE), ATP, NO, vasoactive intestinal peptide (VIP), Tachykinins (TK), Calcitonin gene-related peptide (CGRP), neuropeptide Y (NPY), and substance P (SP) (described in detail elsewhere [4, 5, 7, 12–14]).

### 1.4. Anatomical or Functional Evidence for a Neuroimmune Synapse in the Gut?

Most studies of sympathetic innervation of lymphoid organs employed immunohistological techniques in which the rate-limiting enzyme of norepinephrine synthesis, tyrosine hydroxylase, was detected. These studies, that date back to almost 50 years ago (reviewed in [4, 5]), demonstrated a rich sympathetic innervation of all primary (thymus and bone marrow) and secondary (spleen and lymph nodes) lymphoid organs (discussed earlier, e.g., in [8, 15–17]). Regarding the possibility of a real neuroimmune synapse, electron microscopic studies of the white pulp reveal that sympathetic nerve terminals are in direct apposition to T-cells and adjacent to both dendritic cells and B-cells [16], with the neuroimmune junction being approximately 6 nm wide [18], in contrast to a typical CNS synapse that is approximately 20 nm wide. The close proximity of sympathetic nerve terminals to immune cells provides a mechanism not only for specific targeting of norepinephrine release to immune cells, but also for the containment of neurotransmitter release and the interference with immunological synapse-related processes. Hence, concerning the spleen, such a complete “circuit” appears to exist between the immune system and sympathetic neurons and is most likely projected to intestinal follicles as well. 

### 1.5. Microbial Activity on the Gut-Brain Axis

Apart from direct interaction between nerves and immune cells, (stress-induced) changes to the microbiome may affect the brain and behavior. Studies suggest that inflammatory cytokines disrupt brain neurochemistry and make people more vulnerable to anxiety and depression, possibly explaining the observation that relatively more of IBD or IBS patients also suffer from anxiety and depression. 

As indicated above, the gut-brain axis is a communication system that integrates neural, hormonal, and immunological signaling between the gut intestinal microbiota and its metabolites with a potential route through which to access the brain. This communication system is bidirectional, enabling the brain to influence gastrointestinal functions (such as motility, secretion, and mucin production) as well as immune functions. The influence of gut bacteria on health has received increasing attention, and it is perhaps not surprising that a growing body of the literature focuses on the impact of enteric microbiota on brain and behavior. This communication acts vice versa; there is a growing appreciation that microbiota influence CNS function, and the CNS influences the microbiota composition through its effects on the gastrointestinal tract. How such communication occurs is not fully understood and probably involves multiple mechanisms. Emotional factors such as stress or depression influence the natural history of IBD and IBS via the gut-brain axis. These conditions are also associated with dysbiosis [19, 20]. A likely explanation or an explaining factor, is the fact that stress dramatically affects the disease course of both IBD and IBS. Stress influences the integrity of the gut epithelium and alters gut motility, secretions, and mucin production, thereby altering the habitat of resident bacteria and promoting changes in microbial composition or activity. In addition, stress-induced release of catecholamines into the gut which has a great impact on intestinal immunity, immune cells responses, and migration upon microbial challenge. 

An example of how the microbiome affects behavior is given recently in elegantly performed mouse models of microbiome transfer on genetically different mice strains roared under germ-free conditions [21, 22]. It is known that mouse strains differ in their behavioral phenotype; BALB/c and NIH Swiss mice, for instance, differ greatly in their natural behavior; BALB/c mice are averse and cautious in nature and less exploratory while NIH Swiss mice display a high exploratory drive. It is shown that these behavioral treats are totally determined by commensal flora, as mice colonized with microbiota from the other strain exhibited a behavior profile similar to the donor [23, 24]. Interestingly, the recolonization as well as any of the methodologies used in these studies induced measurable changes in gut inflammation or changed levels of intestinal serotonin (5-HT), NE, or dopamine. In other studies using subdiaphragmatic vagotomy or chemical sympathectomy before antimicrobials suggest that vagal and sympathetic pathways are not involved in gut-brain communication in this experimentally induced dysbiosis model of altered behavior. Hence it is likely that these microbiotic changes in behavior involve molecular pathways beyond these systems, such as the central nervous system expression and modulation of Brain-Derived Neurotrophic Factor (BDNF) [25]. The role for BDNF could be deduced from experiments in which administration of oral antimicrobials to SPF mice altered the composition of the microbiota, accompanied with an increased exploratory behavior and hippocampal expression of BDNF [25].

## 2. Sympathetic Regulation of the Mucosal Immune System

### 2.1. Sympathetic Nerves Found in GALT

With respect to NE, the sympathetic nervous system (SNS) constitutes the largest and most versatile component of the autonomic nervous system. In the periphery, the sympathetic neurotransmitter norepinephrine (NE) is released nonsynaptically, that is, from varicose axon terminals, without synaptic contacts. Thus, ARs on immune cells are targets of remote control, and NE may act as a modulator of the sympathetic-immune interface. Interestingly, as in other lymphoid tissues, serotonergic enterochromaffin cells [26] and intestinal mucosal mast cells [27, 28] lying immediately under the epithelium are selectively associated with enteric nerves [14]. This is interesting in the light of the role for mast cells in the pathogenesis of functional bowel disease such as Irritable Bowel Syndrome [29] where mast cells were shown to be associated with nerve endings. As indicated Section 1.4, a suggestive interaction between mucosal immune tissue and adrenergic nerves is given by anatomical evidence among functional evidence. In GALT including Peyer's patches that represent clusters of lymphoid nodules in the intestines, noradrenergic fibers run profusely into the dome region of the follicles and lamina propria. Here, fibers follow small vessels and branch freely in the parenchyma among fields of lymphoid cells, usually not in association with blood vessels [8]. Nerves that predominate in T-cell zones of lymphoid follicular nodes, contain neuropeptides and the sympathetic neurotransmitter NE.

### 2.2. Stress and Intestinal Immunity: Activation of the HPA Axis

The central nervous system (CNS) interacts with the intestine through what is known as the brain-gut axis; comprising of the hypothalamic-pituitary-adrenal (HPA) axis and the autonomic nervous system (ANS). In turn, the ANS consists of the parasympathetic and sympathetic nervous systems and the ENS located within the intestinal wall. Both vagal and spinal sensory neurons terminate at various points within the gut wall including the muscularis and the mucosal epithelium and play an important role in the transfer of information from the CNS to the ENS. The neuroendocrine system and the immune system have been considered as two autonomously acting networks. The neuroendocrine system responds to external stimuli such as temperature, pain, and stress, whereas the immune system responds to exposure to bacteria, viruses, and tissue trauma. It is now increasingly recognized that these two systems act in close synergy; the immune system is regulated by the CNS, in response to environmental stress, either directly by the ANS or via activation of the HPA axis. This intimate bidirectional network is based on the fact that the immune and neuroendocrine systems share ligands such as neuropeptides, hormones, cytokines, and the respective receptors. Various neuropeptides are released at the peripheral endings of sensory and efferent nerves in response to various invasive and inflammatory stimuli. Neuropeptides, such as opioids released during inflammation, possess anti-inflammatory and antinociceptive properties, which render them potential candidates to treat the unwanted immune responses that occur in inflammatory and autoimmune disorders, by tuning immune homeostasis in a cytokine-like manner.

### 2.3. Adrenergic Receptor Expression on Gut Immune Cells

NE acts on adrenergic receptors which are G protein-coupled receptors and includes nine different gene products: three *β* (*β*1, *β*2, and *β*3), three *α*2 (*α*2A, *α*2B, and *α*2C) and three *β* (*β*1, *β*2, and *β*3) receptor subtypes. At low concentrations (10^−9^ to 10^−7^ M), NE binds to *α*-adrenoreceptors leading to decreased cAMP levels. At high concentrations (10^−7^ to 10^−5^ M) NE binds to *β*-adrenoreceptors, increasing cAMP levels. The ARs are G protein-coupled receptors (GPCRs) that are composed of *α*1 (A, B, and C), *α*2 (A, C, and D), and *β* (*β*1, *β*2, and *β*2) that are coupled with Gq, Gi, and Gs proteins, respectively. Extensive evidence is provided that dendritic cell functions may be tuned by the sympathetic nervous system via the local release of norepinephrine (see for review the literature [26, 30–32]). In the presence of antigens or microbial products, such as agonists for Toll-like receptors 2 and 4, norepinephrine inhibits dendritic cell migration, antigen presentation, and T-helper cells type 1 priming. This effect, which is mainly mediated by beta-adrenergic receptors in DC and interleukin-10 production [26], limits potentially damaging reactions and is functional in shaping the appropriate humoral immune response to extracellular pathogens that need antibodies to be neutralized. In addition, the response to contact sensitizers seems to involve a modulation of the local sympathetic activity. Thus, the sympathetic nervous system may play a crucial role in modulating DC function during the innate phase of the immune response. This evidence has many pathophysiological implications and offers new tools for modulating the immune response.

### 2.4. Adrenergic Regulation of Myeloid Cell Function

Myeloid dendritic cells (DCs) play a key role in determining the appropriate immune response to invading pathogens and tolerance to self-antigens. Besides the type of invading pathogen and its route of entry into the organism, other local microenvironmental factors seem to play a role. 

Myeloid cells, in particular DCs, have been described to express cholinergic as well as adrenergic receptors of several subclasses. DCs express *β*-ARs, and *α*1- and *α*2-ARs, as described by others, reviewed in [4, 5]. The ARs mediate the functional effects of epinephrine and NE by coupling to several of the major signaling pathways modulated by G proteins. Immature as well as mature bone marrow-derived DCs express the mRNA coding for the *β*1b-, *β*2-, *β*1-, *β*-2b, and *β*2C-ARs in the mouse system [26, 33]. As for many other neurotransmitters or their receptors the functional role on antigen-presenting cells is not entirely clear, as functional responses to NE may be dose, time, and AR dependent. Cholinergic receptors are functionally expressed on DCs [34], but recent data imply that the potential of AR to modulate DC APC function outweighs the cholinergic receptors. In general, NE pretreatment has been described earlier to suppress production of proinflammatory cytokines, including IL-12 and TNF-*α*, whereas it enhances the production of IL-10, similar to cAMP-elevating agents, such as forskolin [31, 35]. 

NE seems to exert both a chemotactic and chemokinetic activity on immature DCs influencing their antigen-presenting capacity. Immature DCs which express *α*1b-ARs are recruited to regional lymph nodes upon activation with NE, thereby facilitating antigen presentation to T-cells and possible induction of tolerance [30]. All together these date suggest an anti-inflammatory role for NE on iDC function resulting in mDC inhibiting Th1 and enhancing Th2 differentiation. Furthermore, short-term exposure of bone marrow-derived DCs to NE at the beginning of lipopolysaccharide (LPS) stimulation hampered IL-12 production and increased IL-10 release. The capacity of NE-exposed DCs to produce IL-12 upon CD40 cross-linking and to stimulate allogeneic Th lymphocytes was reduced. This effect corresponded with a strong Th2 and Treg skewing potential of dendritic cells preexposed to AR beta2 agonists. The overall effect of the sympathetic neurotransmitter NE in innate immunity seems thus that of modulating DCs migration and helper T effector cell priming. Hence the role of the DCs ARs would be to limit the inflammatory response to a given pathogen and to modulate the type and strength of the adaptive response. With respect to the above-described adrenergic modulation there are two comments to be made.

First, although many in vivo studies are performed, most of this data derive from bone marrow-derived DCs, and the functionality of these observations are somewhat less well documented [30]. 

Second, the transmitters such as acetylcholine or catecholamines are secreted not only by local autonomic nerve fibers, but also by immune cells under inflammatory conditions suggesting that at least in models of experimental colitis, local immune cells regulate catecholamine release in an autocrine or paracrine manner.

### 2.5. In Vivo Relevance of Adrenergic Receptor Activation

In order to study the role of sympathetic immunoregulation strategies to deplete adrenergic innervation, chemical strategies for sympathectomy using dopamine analogues (6-OH Dopamine) and alternatives thereof (see for details, e.g., [36] and early description [37] as well as others) have been well characterized, also in the context of colitis. Catecholamines are synthesized from tyrosine that is transported into the noradrenergic ending or varicosity by a sodium-dependent carrier. Tyrosine is converted to 3,4,dihydroxyphenylalanine (L-DOPA) (the rate-limiting step in the NE synthesis) by the enzyme tyrosine hydroxylase (TH) and finally to dopamine (DA), and a carrier that can be blocked by reserpine transports dopamine into the vesicle. Dopamine is converted to NE in the vesicle by dopamine-*β*-hydroxylase. In the adrenal medulla, NE is further converted to epinephrine. TH- and dopamine-*β*-hydroxylase immunostainings are often used as specific markers for nor-adrenergic innervation in various organs. Chemical sympathectomy using a blocker of TH, 6-hydroxy-dopamine (6-DOPA) resulted in a decreased damage score and improved histology compared with controls in TNBS colitis rats.

In disease models for IBD, contrasting roles for the anti-inflammatory effect of the SNS arise. In chronic DSS colitis and the IL-10-knockout model of IBD, sympathetic denervation using 6-DOPA exacerbated disease [38, 39]. During acute DSS colitis and TNBS-induced colitis, sympathetic denervation decreases inflammation. These data suggest that the SNS exerts proinflammatory effects at the beginning of tissue inflammation while it confers anti-inflammatory effects in the chronic phase of inflammation Table 1.

## 3. Parasympathetic Control of the Gut Immune System

### 3.1. The Anti-Inflammatory Activity of the Efferent Vagus Nerve and Intestinal Immunity

Functional evidence demonstrated that the vagus nerve displays a regulatory role for immunity via direct preganglionic innervation (directly) or indirectly by affecting postganglionic fibers. This notion is originally deduced from the neural regulation of the neurohormonal hypothalamus-pituitary-adrenal (HPA) axis with its associated glucocorticoid production and for induction of fever. Afferent vagus nerve activity in response to LPS or IL-1*β*-administration in the periphery causes efferent vagus nerve signaling to the thymus and increased activity in the splenic nerve [40, 41]. Subdiaphragmatic vagotomy abolished the effects on adrenocorticotropin of intraperitoneal administration of LPS or IL-1*β* and blocks IL-1*β*-induced hyperthermia and other centrally mediated effects, including glucocorticoid production. Increased cytokine levels in the periphery and exogenous administration of proinflammatory cytokines also elicit sickness behavior. Reciprocally, blockade of the cytokines IL-1*β*, IL-6, and TNF abolishes this response. These findings were later supported by the observation that vagal ganglia express IL-1 receptors, providing a direct mechanism by which IL-1*β* can directly activate vagal nerve afferent fibers [42]. 

With respect to the efferent (motor) arm of the vagus nerve, the pioneering work by Tracey and coworkers [40, 43] demonstrated that electrical stimulation of the vagus nerve prevents the development of endotoxin-induced shock by reduction of proinflammatory cytokine production, in particular TNF*α* in the spleen [44]. This anti-inflammatory effect could be reproduced in vitro using isolated human macrophage cultures; the release of TNF, interleukin IL-1*β*, IL-6, and IL-18 in response to endotoxin was significantly reduced by acetylcholine (ACh) and nicotine (a prototype nicotinic acetylcholine receptor blocker). Wang et al. identified the *α*7 subtype of the nicotinic acetylcholine receptor (*α*7nAChR) as the main receptor by which splenic macrophages are modulated. The anti-inflammatory effect of VN stimulation is lost in *α*7nAChR knock-out mice, can be blocked by specific antagonists *α*7nAChR, and is mimicked both in vivo and in vitro by *α*7nAChR agonists. In more recent studies ongoing work has indicated the crucial interplay between the vagus nerve and sympathetic (celiac) ganglia in mediating the anti-inflammatory effect of vagus nerve efferent activity [3, 44].

This anti-inflammatory effect is mediated by activation of *α*7nAChRs located on immune cells (in particular macrophages) in response to ACh released by vagal efferent nerve terminals. The discovery of the cholinergic anti-inflammatory vagal efferent pathway introduced the concept of the “inflammatory reflex” [40] by which the central nervous system is capable of sensing inflammation and reflexively modulating the immune response. 

Although it is well established that proinflammatory cytokines and endotoxin stimulate vagal afferents leading to brain stem activation (see for review, e.g., [45]); data supporting activation of motor neurons of the vagal nerve closing the anti-inflammatory loop to the gut are only recent [46]. Anatomical evidence was provided supporting the notion that subtle intestinal inflammation is detected by vagal afferents triggering NTS activation and generating a specific vagal outflow previously shown to modulate the inflammatory response. Retrograde labeling using cholera toxin-b conjugates was used to map the neuronal innervation of the small intestine and the spleen. The retrograde labeling was limited to the circumscribed region of the DMV [46, 47]. CTB-labeled neurons were localized in the lateral part of the DMV observed from −7.32 to −7.76 mm Bregma, as expected from previous anatomical studies. Moreover, it was demonstrated that subtle intestinal inflammation leads to activation of NTS and DMV that is abolished by selective intestinal vagotomy. Importantly, more than 40% of the activated DMV neurons targeted the inflamed intestine [46], supporting the existence of an endogenous vagal “inflammatory reflex” modulating intestinal inflammation. 

In studies focusing on the gut mucosa, perioperative electrical stimulation of the vagus nerve decreases intestinal inflammation due to manipulation surgery and postoperative ileus by inhibiting macrophage activation [48]. Later analyses revealed that this cholinergic anti-inflammatory pathway (i.e., DMV activation) mainly occurs 24 h after surgical insult, that is, once leukocytes have infiltrated the gut muscularis, but not at the earlier stage where chemokines and cytokines are secreted by the resident immune cells. Compatible with this hypothesis is the observation that vagal denervation of the small intestine did not significantly enhance the inflammatory response directly after surgery [46]. 

Given the fact that vagal innervation of the (especially distal) colon is relatively scarce, the immune regulatory action that vagal activity may have to colonic immune responses is different. Nevertheless, given the potent anti-inflammatory effect of the cholinergic innervation, one might assume that the cholinergic tone in the submucosal compartment may have an important impact on mucosal immune homeostasis. In mouse models of colitis, enhanced parasympathetic output is described to be involved in the negative regulation of intestinal inflammation via efferent activity of the vagus nerve [49, 50]. Indeed, studies in vagotomized mice revealed increased susceptibility to develop DSS-induced colitis. In an alternative approach, pharmacological stimulation of nicotinic *α*7 receptors aggravated the colitis course in DSS colitis [51]. Notably, reduced mucosal levels of ACh in a murine model of depression were also associated with a more severe colitis in response to DSS. As adoptive transfer of macrophages from depressive mice induced a similar increased susceptibility to develop DSS colitis [23, 51], macrophages were proposed to be (one of) the target cells modulated by the cholinergic tone in the submucosal microenvironment.

Vagal activity may lead to peripheral release of its principal neurotransmitter ACh, but one should keep in mind that the vagal nerve particularly projects to other postganglionic enteric neurons. In this way the vagus nerve regulates gut immune function and barrier function through neuropeptides and neurotransmitters released by the ENS. The latter would implicate that enteric neurons rather than vagal nerve endings interact with the intestinal immune system. The enteric nervous system forms a dense network of nerve fibers in close vicinity with intestinal immune cells, both in the submucosal (lamina propria) and muscular externa compartments of the intestine. This could imply that vagal signals are amplified by the ENS inducing the substantial release of ACh in the intestinal microenvironment leading to modulation of the immune response. However, the possible release of other immune modulator neurotransmitters by enteric neurones cannot be excluded. Indeed, several neurotransmitters that do not belong to cholinergic or adrenergic class, such as vasoactive intestinal peptide (VIP), Substance P, and others, have been shown to modulate immune cells (detailed in Section 4).

### 3.2. Different Cholinergic Receptor Expression on Gut Immune Cells (Macrophages, Dendritic Cells, and Mast Cells)

**Figure 1:**
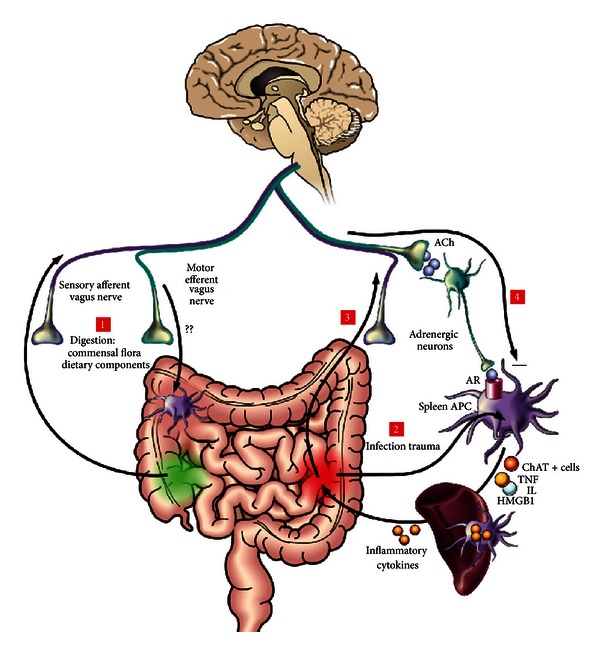
The cholinergic anti-inflammatory pathway depicted. Scheme of the vagus nerve interacting with immune activation at multiple levels following ingestion, infection, and trauma. (1) During digestion, the commensal flora and dietary components activate the sensory afferent vagus nerve, which will transmit the information to the brain. In return, the brain may activate the efferent vagus nerve to modulate gastrointestinal macrophages. (2) The efferent vagus nerve also modulates systemic inflammatory responses through a mechanism involving an intact spleen. Upon infection or trauma, bacterial components or intracellular mediators (HMGB1, heat shock proteins, etc.) activate macrophages to produce proinflammatory cytokines. (3) This will trigger afferent vagus nerve signaling. (4) Central activation of vagal efferent pathways which lead to release of acetylcholine (ACh) and relay on peripheral ganglia to stimulate adrenergic transmitter release (in the spleen) to act on aplenic antigen presenting cells and macrophages. Recent data indicate the generation of ChAT-positive ACh producing immune cells (see text). Interrogation marks indicate that, although macrophages are found in the proximity of cholinergic fibers in the spleen and the intestine, there is currently no evidence demonstrating that parasympathetic neurons, indeed, innervate immune cells in the gut wall. HMGB1: high-mobility group box 1.

**Figure 2:**
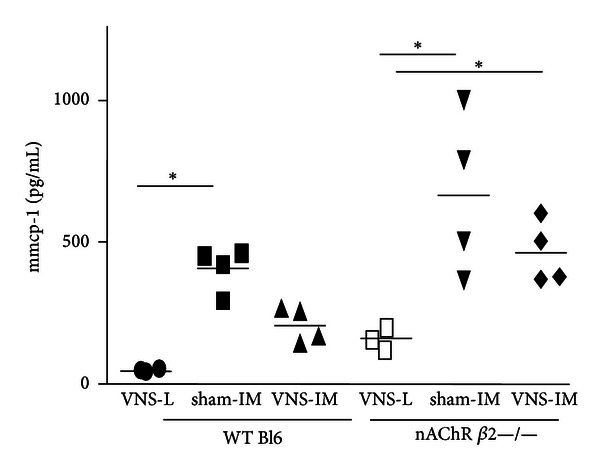
MMCP-1 in peritoneal lavage. Electrical stimulation of the vagus nerve in mice leads to decreased release of mast cell specific products in the peritoneal cavity. Shown are the levels of mouse mast cell specific protease 1 (mmcp-1) in the peritoneal cavity of mice that underwent vagal nerve stimulation of the cervical nerve 30 minutes before laparotomy control (L) or intestinal manipulation (IM) surgery. The effects of VNS depend on activation of the nAChR *β*2 [52], as VNS has no effect on mice deficient in this receptor. Unpublished data, S. A. Snoek, Tytgat Institute, 2012.

**Figure 3:**
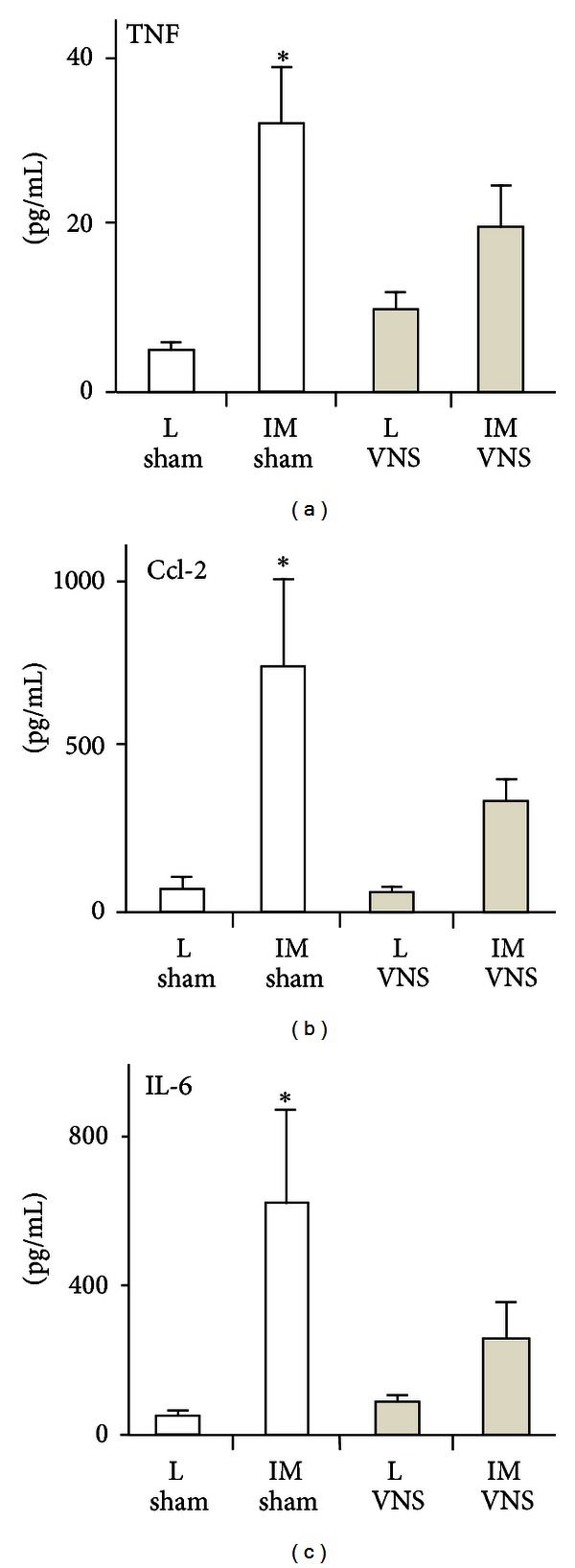
Electrical stimulation of the vagus nerve in mice leads to decreased release of proinflammatory gene products in the peritoneal cavity after surgical manipulation. Shown are the levels of mouse TNF, CCL-2, and IL-6 in the peritoneal cavity of mice that underwent vagal nerve stimulation (VNS) of the cervical nerve 3 h before laparotomy control (L) or intestinal manipulation (IM) surgery. Data are modified from [48], Tytgat Institute, 2012.

Although much attention has been given to macrophages, a multitude of other immune cells (T-cells, dendritic cells (DCs), and mast cells) residing in the mucosa/submucosa carry nicotinic or muscarinic receptors and may be affected by the anti-inflammatory pathway as well. Another important but less studied population of intestinal immune cells are the macrophages located between the longitudinal and circular muscle layer at the level of the myenteric plexus. These resident macrophages play an important role in diabetic induced gastroparesis, postoperative ileus (POI), and LPS-induced septic ileus and seem to represent the gatekeepers of the enteric nervous system or the “little brain of the gut.” Vagal activation by means of electrical probes was shown to prevent muscular inflammation and ileus following intestinal manipulation, suggesting that this subpopulation of macrophages is also under cholinergic control (Figures 2 and 3). By determining the cholinergic tone in the enteric nervous system, the vagal innervation may modulate the intestinal microenvironment (Figure 1). Hence, the set point (balance) of the gut immune system will be affected influencing not only macrophages or DCs, but also theoretically any immune cell carrying cholinergic receptors, that is, T and B lymphocytes, monocytes, and mast cells [27, 28].

The machinery to generate ACh and to express its receptors is not limited to cells of the nervous system (see Section 3.3). The key enzyme choline acetyltransferase (ChAT) is virtually found in every human cell, including immune cells [53]. ACh couples to two distinct ACh receptor subtypes (AChR). Nicotinic ACh receptors (nAChRs) are ligand-gated ion channels consisting of twelve distinct subunits, nAChR*α*2–10 and nAChR*β*2–4 in mice. Muscarinic ACh receptors (mAChR1–5) are G protein-coupled receptors. Murine peritoneal macrophages express nAChR*α*2–7 and 10, nAChR*β*2 and 4, and all muscarinic receptors [54], whereas human primary macrophages express nAChR*α*2,4, and 7, nAChR*β*2 and 4, and mAChR1,4, and 5. Transcripts for nicotinic acetylcholine receptors (nAChRs) subunit *α*7, beta2, and alpha4 have been detected in multiple inflammatory cell types, including macrophages derived from various tissues and intestinal lamina propria [43, 55, 56]. The finding of distinct nAChR subtypes expressed in immune cells suggests that nicotine may differentially affect distinct inflammatory cells with its specificity based on receptor affinity for ACh, as is the case in neurons (reviewed in [57]).

Most evidence points towards a crucial role for the *α*7nAChR homopentamer in the cholinergic regulation of macrophage activity [43]. ACh and nicotine effectively attenuate macrophage activation by decreasing the production of a variety of proinflammatory mediators, including HMGB1 [43], TNF, IL-1*β*, and IL-6 (a.o. reviewed in [58]). Anti-inflammatory actions in monocytes/macrophages seem to be predominantly facilitated via nAChR*α*7. Activation of this receptor inhibits proinflammatory cytokine transcription via distinct mechanisms. Stimulation of macrophage nAChR*α*7 inhibited nuclear translocation of NF-*κ*B, likely by blocking degradation of the NF-*κ*B inhibitor I-*κ*B [59] or via activation of signal transducer and activator of transcription (STAT)3 in murine macrophages in vitro [48, 60].

In addition, the *α*7 nAChR subunit mediated anti-inflammatory effects in vivo. The *α*7nAChR subunit is expressed by macrophages, and its expression is crucial for the anti-inflammatory effect of vagal nerve signaling. Moreover, nicotine exerts anti-inflammatory effects on macrophages that can be counteracted by selective *α*7 antagonists. Nicotinergic agonists, selective for the *α*7 nAChR, have proven effective in reducing macrophage cytokine production and inflammation in animal models of pancreatitis, DSS-induced colitis, and intestinal ileus (reviewed a.o. in [61]).

Notably, *α*4*β*2nAChR rather than *α*7, activation enhances the phagocytic potential in mouse macrophages while, despite enhanced phagocytosis, the activation of NF-*κ*B activity and proinflammatory cytokine production in these cells is inhibited. In addition, electrical stimulation of the vagus nerve protects the epithelial permeability disturbances for luminal bacteria and stimulates phagocytosis by F4/80^+^CD11^b+^ macrophages residing in the intestinal mucosa. Supporting evidence for an anti-inflammatory vagal activity, transmitted via macrophage nAChR*α*7, emerged from CNS disorders. Patients with traumatic brain injury suffer from immune paralysis due to an increased vagal tone [62]. Along this line, patients suffering from inflammatory bowel disease (IBD) frequently show signs of depression. Ghia et al. observed reactivation of quiescent colitis in a murine model of depression, which was associated with impaired cholinergic inhibition of proinflammatory cytokine secretion by macrophages through nAChR*α*7 [23]. Besides nAChR*α*7, other AChRs have also been implicated in macrophage biology. ACh and vagus nerve stimulation enhanced the capacity of murine macrophages to phagocytose bacteria. Again, ACh reduced NF-*κ*B activation and proinflammatory cytokine production, while increasing the release of IL-10 [61]. Enhanced phagocytosis and increased IL-10 production resulted from *α*4*β*7nAChR activation [56].

Taken together, vagus nerve activity can enhance macrophage bacterial uptake via activation of the *α*4*β*7nAChR, while reducing inflammatory cell activation via differential nAChRs including that of the *α*7 subtype. 

### 3.3. Nonneuronal Acetylcholine Production in the Gut (Epithelial Cells, T-Cells, and Others): The Work on Spleen Cells

As already indicated by the widespread expression of AchR subtypes among intestinal nonneuronal cell types, cholinergic signaling is not restricted to neurons. As indicated, ACh can be produced by a mechanism involving ChAT, which is expressed in both neuronal and non neuronal tissues. Importantly, it is shown that these enzymes ChAT and OCT transporters for the uptake of choline, are present in colonic epithelial cells. Other than the epithelium [63], lymphocytes [54, 64] have been extensively reported to express ChAT and produce measureable quantities of ACh. Kawashima et al. have previously reported that 60% of ACh in the blood is produced by mononuclear lymphocytes. In other organs such as the spleen; immune cells display a tightly regulated cholinergic activity that seems dictated by inflammatory activation [3] (reviewed a.o. in [1, 41]). 

Another role of the cholinergic system, not directly involving AChRs, was uncovered during endotoxin tolerance. LPS treatment reduced the expression and activity of ACh esterase (AChE) via miR-132 in human and murine macrophages [65]. The impact of miR-dependent regulation during inflammation was substantiated by LPS treatment of mice carrying a 3′UTR-Null-AChE transgene. These mice displayed a dysregulated body temperature after endotoxin challenge and isolated bone marrow- (BM-) derived macrophages failed to inhibit inflammatory cytokine production after ACh administration [65].

 In an intestinal context, previous studies have implicated that a variety of nonneuronal cell types have the capability to produce Ach, including epithelium, immune cells, and fibroblasts [53, 66]. Thus, ACh can no longer be considered simply as a neurotransmitter but rather as a ubiquitous intercellular messenger that is likely to be important in integrating many different aspects of intestinal physiology in health and disease. In fact, nonneuronal cells, including those of the heart, lungs, vasculature, immune cells, bone, and brain, have been known for many years to have the capability to synthesize and release ACh. In the intestine, nonneuronal ACh release was identified more than a decade ago [63]; nonetheless its physiological relevance is still poorly defined. Hence, ACh production by non neuronal cells might play an important role in regulating actions of cells that are not juxtaposed with cholinergic neurons such as epithelial cells and immune cells. This notion may have important implications and stress the role of cholinergic nonneuronal signaling to maintain immune homeostasis.

Although a direct evidence for the role of non neuronal cells producing ACh in the gut is lacking, recent studies on ACh producing immune cells in the spleen provide exciting insights into their potential role in inflammatory diseases of the gut. Recently, the group of Tracey has recently isolated and characterized the role of ChAT-positive lymphocytes in a model of systemic inflammation [3]. The authors identified a subpopulation CD4+ CD62L+ T-cells as ChAT-positive T-cells that secrete ACh, express *β*-adrenergic receptors, and are located adjacent to adrenergic nerve endings in the spleen. These T-cells were reported to be responsible for downregulating TNF*α* production following vagal nerve stimulation during sepsis. Similar observations done somewhat earlier already indicated the involvement of epinephrine and *β*-adrenergic receptor activation on T-cells in mediating the vagal anti-inflammatory response [44, 60, 67]. Whether lymphocyte populations arriving in the gut during chronic inflammatory state are derived from the spleen remains debatable. We can anticipate that the resident immune cell populations in GALT have a similar subsets of immune cells as described in the spleen. As discussed in Section 2, GALT and in particular PP's are extensively innervated by sympathetic nerve fibers, and in inflammatory conditions be proof to be an important source of ACh producing immune cells.

## 4. Noncholinergic Nonadrenergic Receptors: Relevance for Gut Immunity

Neuropeptides are considered key mediators in the communication between neurons (in particular sensory neurons) and effector cells (smooth muscle, glands, and immune cells) and exhibit a variety of functions in the gastrointestinal tract. Neuropeptides are involved in secretion of salivary, gastric fluids and intestinal fluids, and electrolytes and G protein-coupled receptors (GPCRs) that bind neuropeptides represent potentially useful peptide therapeutics. Besides the action on motor function of the gut, neuropeptides also function as cotransmitters of enteric cholinergic neurons, increase enteric neuron excitability, and consequently induce the release of enteric neurotransmitters, including acetylcholine [80]. Neuropeptides are increasingly recognized as potent modulators of the immune response, which is underscored by the fact that, in addition to (afferent) neurons, several immune cells produce neuropeptides. Here a selection of neuropeptides will be discussed.

### 4.1. The Role of Substance P in Intestinal Immunity

SP is an 11 amino acid peptide released from both the central and peripheral endings of primary afferent neurons. The effects of SP are mediated by 3 GPCR neurokinin- (NK-) 1, -2, and -3. The NK-1 receptor is the high-affinity receptor for SP, while NK-2R and NK-3R bind with much lower affinity to this peptide [81, 82]. The gut is one of the most abundant sources of SP in the body, and SP is particularly synthesized by enteric cholinergic motor neurons that project to the longitudinal and circular muscle of the intestine. Besides neurons, other sources of SP in the gut are enteroendocrine cells, colonic eosinophils, lamina propria macrophages, and colonic glia [81, 83]. The NK-1R was found to be distributed in all layers of the human ileum and colon smooth muscle cells of the muscularis mucosae and propria, both in the longitudinal and circular muscle and neurons of the myenteric plexus. SP acts via the NK-1R present on multiple local cells, including intestinal nerves, cells of the muscular wall of submucosal blood vessels, intestinal epithelial cells, and immune cells such as macrophages, mast cells, and neutrophils [84, 85].

*SP Expression during Intestinal Inflammation.* The involvement of SP-NK-1 interaction in pathophysiology of intestinal inflammation has been shown in both animal models of IBD and in human disease. SP levels are increased in the peripheral blood of rats with dextran sulfate sodium (DSS) colitis and decreased in the entire colon early after intracolonic TNBS [82]. Increased SP expression has been observed in both tissue and nerve fibers in the colons of patients with UC and is correlated with disease activity. However, in UC patients with severe disease activity, the density of SP immunoreactive nerves appears to be decreased. Other studies, however, failed to demonstrate altered SP immunoreactivity in the colonic or rectal mucosa in UC patients versus controls [82]. Increased NK-1R expression is observed in tissues of both CD and UC patients on inflammatory cells of the lamina propria, epithelial cells lining the mucosal surface and crypts, lymphoid aggregates, small blood vessels, and enteric neurons. In both diseases enhanced expression of NK-1R is observed not only in surgical specimens collected from the center of inflammation, but also in samples obtained from macroscopically uninvolved areas [85].

*Modulating SP in Intestinal Disease.* A functional role for SP in intestinal disease is described. Smooth muscle function disturbances represent an important consequence of inflammation and contribute to the pathophysiology of the most common clinical symptoms of IBD, such as abdominal pain, cramps, and diarrhea. NK-1R participates in basic colonic responses, such as chloride secretion, gut permeability, and modulation of inflammation. Activation of both NK-2 and NK-3 receptors affects motility responses in the GI tract, but there is little evidence that these receptors are involved in neuroimmune interactions. However, NK-1R antagonists have proven to ameliorate inflammation in TNBS- and DSS-induced colitis mouse models. It was shown that treatment with the NK-1R antagonist CP-96345 reversed inflammation-induced alterations of smooth muscle contractility, thereby further contributing to amelioration in intestinal disease. Furthermore, in addition to its proinflammatory effects, activation of NK1R also enhances chloride secretion thereby inducing diarrhea and further worsening disease. Mice genetically deficient in the NK-1R are protected from the secretory and inflammatory changes induced by *C. difficile* toxin A, demonstrating a major requirement for SP receptors in the pathogenesis of inflammatory diarrhea [77, 85]. Further, it was demonstrated by using selective nonpeptide NK-lR (SR 140333), NK-2R (SR 48968), and NK-3R (SR 142801) antagonists, NK-lR and NK-2R but not NK-3R antagonists reduced colonic inflammation in a rat model of TNBS-induced colitis [76]. In addition, chronic administration of the nonpeptide NK-1R antagonist CP-96345 significantly reduced the disease activity in experimental DSS colitis models [86].

Together, the results obtained from studies in animal models of intestinal inflammation show that NK-1R activation in the intestine enhances intestinal inflammation, reduces smooth muscle function, and increases inflammatory diarrhea. This suggests that antagonizing NK-1R might provide a novel therapeutic approach in the treatment of intestinal inflammation. An analysis of the literature to date reveals the presence of more than 1500 patent applications for NK-1R antagonists [77]. All of the NK-1R antagonists tested so far in humans appear to be well tolerated. Some antagonists have been used in clinical trials as antiemetic and for the treatment of pain and anxiety/depression. So far, NK-1R antagonists have not been applied in human studies regarding intestinal inflammation. 

### 4.2. Vasoactive Intestinal Peptide (VIP)

VIP plays a significant role in intestinal physiology such as regulation of the peristaltic reflex in the circular smooth muscle layer, and VIP-deficient mice show anatomical and functional abnormalities in the gut. VIP is a 28 amino acid neuropeptide produced in the central and peripheral nervous systems, including nerves within the GI tract. The source of the VIP in the gut derives largely from intrinsic enteric VIP-containing neurons in the myenteric and submucosal ganglionic plexuses and from extrinsic parasympathetic autonomic nerves and sensory fibers [7, 87, 88]. Although primarily localized to neurons, immunomodulatory cell types including T-cells [89, 90], B-cells, mast cells, and eosinophils also produce VIP [91].

*VIP Expression during Intestinal Inflammation.* The broad spectrum of biologic actions in which VIP is involved also includes immunomodulatory functions. VIP has anti-inflammatory and immune cell-modulating activities [82], including inhibition of the leukocyte migration and stimulation of IgA production by lamina propria B lymphocytes [92]. VIP acts by activation of pituitary adenylate cyclase-activating polypeptide (PACAP)/VIP mutual receptors VPAC1 and VPAC2. VPAC1 and VPAC2 are expressed abundantly throughout the intestine. Although VPAC1 and VPAC2 are both highly expressed throughout the GI tract, the VIP receptor subtypes exhibit complementary expression patterns in the small intestine. VIP exerts its effects by binding to two different receptors, namely, VPAC1 and VPAC2, which belong to the class II family of guanine nucleotide-binding protein- (G protein-) coupled receptors.

*Modulating VIP in Intestinal Disease.* The different functions mediated by VIP depend on the expression pattern of the various types of immune cells. Dendritic cells both in vitro generated human monocyte-derived DCs and murine bone marrow-derived DCs, and DCs isolated from Peyer's patch express the VIP receptors VPAC1 and 2 [92]. VIP released by peptidergic nerve fibers or immune cells in inflammatory conditions is able to alter the differentiation and activation state of DC.

Immature DCs express high levels of MHC class II but low levels of the costimulatory molecules CD80/86, and its upregulation requires TLR triggering. However, Gomariz showed that iDC treated with VIP upregulated CD86 expression without Toll-like receptor ligands [93]. This semimaturation resulted in enhanced iDC capacity to induce proliferation in T-cells. In conjunction with these data, human monocytes and murine bone marrow DCs differentiated in the presence of VIP acquire a tolerogenic phenotype, characterized by expression of low levels of the costimulatory molecules CD80/86 and CD40, production of low levels of proinflammatory cytokines, and high levels of IL-10 after maturation.

Migratory capacity of DC to local lymph nodes is mediated by the chemokine receptor CCR7. VIP dose dependently downregulates CCR7 expression on DC thereby inhibiting migration of mature DC to local lymph nodes. Several reports have recently proposed the use of VIP tolerogenic DC to induce antigen-specific Treg cells ex vivo and restore immune tolerance in autoimmune disease [94]. VIP-treated—or transduced—DCs injected at the onset of disease in a TNBS colitis model ameliorated the detrimental effect seen in control-treated mice. This was reflected by a downregulated Th1 (TNF-*α*, IFN-*γ*, IL-6, IL-1*β*, and IL-12) cytokine response of immune cells and stimulated IL-10 and TGF-*β* production in TNBS-induced colitis [95, 96]. 

In human studies, the expression of VIP in colonic nerves and rectal tissue may be subject to disease activity in IBD. However, the reported data are conflicting; in CD patients VIP content was increased as compared to UC patients or controls [82], while, in contrast, a decrease in immunoreactivity for VIP in the mucosa of UC patients was observed as compared to control patients [97]. At the VIP receptor level the proportion of VPAC-1 positive cells among CD3-positive T-cells in the lamina propria was significantly higher than in CD patients and controls [90]. Furthermore, the proportion of VPAC-1 positive cells among tissue macrophages was significantly higher in patients with UC and CD than in controls. Hence, especially given the specific changes of VIP seen in CD and UC, these observations may indicate a role for VIP in the disease course, although a change in VIP tissue content most likely represents a phenomenon secondary to the inflammation.

Even though several studies show that VIP administration reduces clinical symptoms and significantly affects cytokine profiles in mouse models of experimental colitis, VIP itself is not useful as a therapeutic agent, because of its low stability in the circulation and low oral availability due to gastrointestinal peptidases. Therefore, extensive studies are conducted to design stabile VPAC1 and VPAC2 receptor agonists and antagonists, possibly via development of nonpeptide small molecules acting at VIP receptors. 

### 4.3. Neuropeptide Y and Peptide YY

Neuropeptide Y (NPY), peptide YY (PYY), and pancreatic polypeptide are structurally related peptides that primarily function as neurotransmitter and gastrointestinal hormone, respectively. NPY is a 36 amino acid peptide neurotransmitter widely expressed in the peripheral and central nervous system. NPY has been associated with a number of physiologic processes in the brain, including the regulation of energy homeostasis, memory, and learning. The main effect is increased food intake and decreased physical activity. More recent evidence also implicates NPY in immune processes [98], especially via activation of the Y1 Receptor [78].

*PYY and NPY Expression during Intestinal Inflammation.* To date, five NPY highly conserved Y receptors have been cloned: Y1, Y2, Y4, Y5, and Y6 [98]. These receptor systems are not only activated by NPY but share affinity with PYY and pancreatic polypeptide. The latter two have similar affinities for all Y receptors. The receptor protein that is activated by NPY is a metabotropic G protein-coupled receptor in the rhodopsin-like GPCR family coupling to G_i_, thereby inhibiting adenylate cyclase. The Y-receptor proteins contain seven membrane spanning domains, and all have been identified in human and rodents, except for Y6 receptor, which is only functional in mouse and rabbit and even absent from rat [99]. Subtypes Y1 and Y5 have known roles in the stimulation of feeding, while Y2 and Y4 seem to have roles in appetite inhibition (satiety).

*Modulating NPY in Intestinal Disease.* The sympathetic regulation of immunity is not only mediated by catecholamines but also involves neuropeptide Y (NPY), as an additional postganglionic neurotransmitter of the sympathetic nervous system that is shown to modulate various immunological functions in vitro and in vivo. NPY is a typical example of the link between the sympathetic nervous system and the immune system, as NPY releasing sympathetic nerves innervates lymphoid organs, and several immune cell types express NPY or its Y1R [79, 100]. In the rat, mononuclear leukocytes were shown to express NPY transcript and protein at high levels (comparable to those found in ganglia), and interestingly its expression is tightly downregulated after immune cell activation [101].

The effects of NPY and Y1R on the immune system are quite pleiotropic, ranging from effects on cytokine release to cell migration and plasma extravasation [79, 100]. NPY may affect immune cells directly via activation of the Y1R or indirectly via modulation of the release of Substance P, (central) release of serotonin or by modulation of catecholaminergic receptor activation [100]. In addition, injection of NPY in local areas in the brain (paraventricular nucleus) resulted in an acute release of corticotrophin-releasing factor (CRH) in the brain, proving that NPYergic activity directly stimulates the release and synthesis of CRH. As indicated above, CRH generally acts as a proinflammatory stimulus stimulating the immune cell activity. 

Genetic ablation of NPY [102] or the Y1R [103] has been shown to result in protection against acute DSS-induced colitis, as indicated by reduced weight loss and inflammation of colon tissue [103]. The phenotype of NPY- and Y1R-deficient mice has revealed that the NPY/Y1R activation is not restricted to the modulation of reactivity of T-cells directly, but is rather part of a fine-tuned system capable of distinct regulation of the potency of antigen-presenting cells (APCs) to induce T-cell reactivity [79]. Indeed, a reduced antigen uptake and production of IL-12 and TNF following TLR ligation were observed in macrophages and dendritic cells lacking NPY. The cellular mechanism by which APC function is affected by NPY or Y1R activation is currently not known, but NPY may affect cellular function in an autocrine fashion.

**Figure 4:**
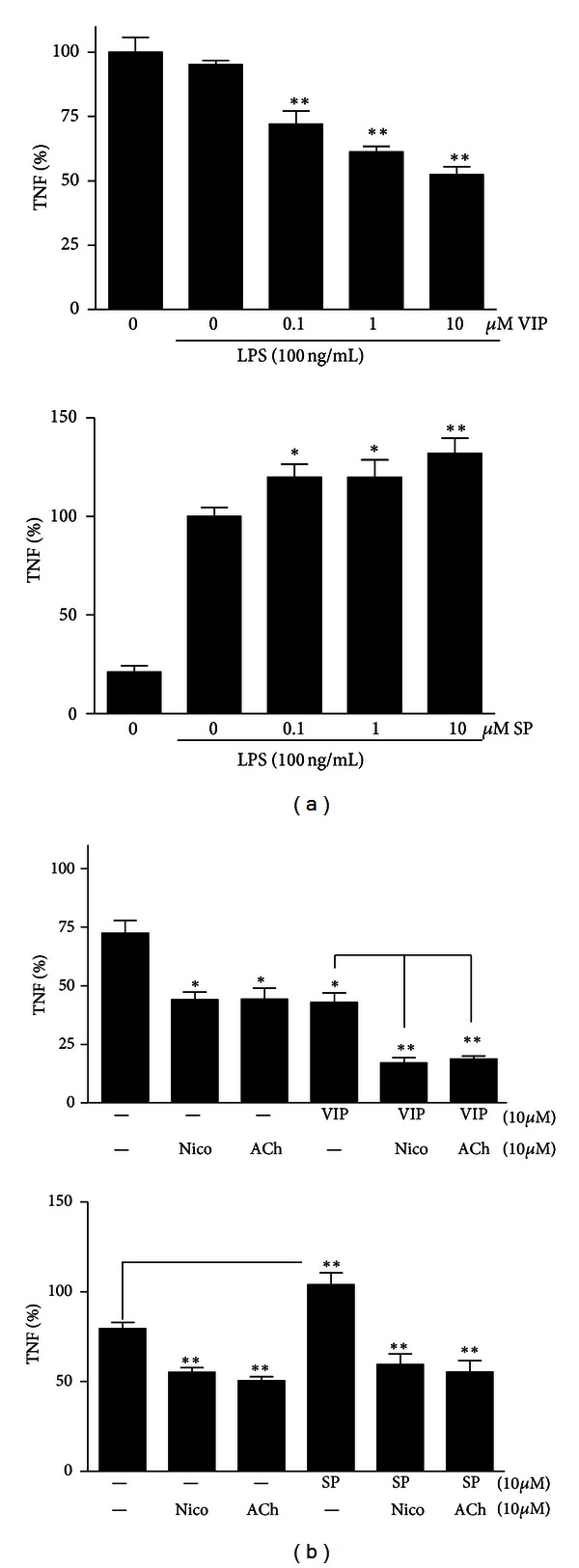
VIP/SP and ACh/nicotine modulate inflammatory mediators in a cumulative manner. (a) RAW peritoneal macrophages were incubated with a dose range of VIP (0–10 *μ*M) for 30 min prior to 3 hrs LPS stimulation (100 ng/mL). TNF production was measured with ELISA. (b) cells were pretreated with 10 *μ*M ACh/nicotine for 30 min, then stimulated with 10 *μ*M VIP or SP, and followed by 100 ng/mL LPS challenge. TNF levels were measured after 3 hrs and depicted as % of vehicle-treated cells. Data are mean s.e.m. of three independent experiments done in triplicate. *P < 0.05; **P < 0.01. E.P. van der Zanden et al., Tytgat Institute AMC, unpublished 2012.

*Interaction between Neuronal Regulation Systems.* Clearly, vagus nerve stimulation may also affect immune cells via postganglionic mechanisms involving alternative neurotransmitters, such as neuropeptides. During the last decade, neuropeptides are increasingly recognized as modulators of the immune system and can play a crucial role in the pathogenesis of inflammation. Multiple studies have demonstrated that cholinergic agonists can interact with immunomodulatory actions of VIP and SP (Figure 4). ACh is the principle neurotransmitter of the vagus nerve, but dorsal motor nuclei of the vagus nerve may also express neuropeptides. Hence, the efferent vagus nerve may be not solely cholinergic, but also peptidergic. Since the half-life of ACh in tissue is far shorter than 1 s, neuropeptides released from cholinergic nerve endings rather than ACh would be expected to play an important role in anti-inflammatory effects observed after VNS. In other intestinal organs, such as the gallbladder, it is already shown that VIP is released upon efferent vagus stimulation [104].

In support of this hypothesis, coapplication of VIP and ACh or nicotine reduces inflammatory mediator production in RAW macrophages in a cumulative manner.

The mechanism via which vagus nerve-released ACh can cooperate with VIP to reduce inflammation is still unclear (illustrated by our preliminary experiments shown in Figure 4).

Gonzalez-Rey and Delgado demonstrated that the anti-inflammatory effect of VIP on RAW cells is mediated via two intracellular pathways: affecting both NF-*κ*B binding and the composition of the cAMP responsive element binding complex (CREB/c-Jun) [74]. The working mechanism of ACh ultimately involves modulation of STAT3 pathways [105, 106] and prevention of NF-*κ*B p65 transcriptional activity [107, 108], following nAChR activation (multiple studies, reviewed in [74]). Possibly, VIP and ACh act synergistic in the inhibition of nuclear translocation of NF-*κ*B. Alternatively, the cross-talk between VIP and ACh could be extracellular instead of intracellular. This is supported by the finding that, in isolated parasympathetic neurons of rat ganglia, VIP selectively increased the affinity of nAChR for their agonists, thereby potentiating ACh-evoked whole cell currents in rat cholinergic neurons. Otherwise, it is possible that ACh modulates VIP and SP receptor expression which renders cells more susceptible to immunomodulation. Finally, studies in rodents have demonstrated that VIP, SP, and ACh can be locally produced by inflammatory cells such as macrophages, lymphocytes, and dendritic cells. Possibly, the nAChR-mediated effects of ACh could be potentiated in presence of locally released VIP.

## 5. Future Perspectives and Clinical Implications

### 5.1. Summary and Future Prospects

The potential of several neurotransmitters to modulate the course of intestinal inflammation via direct or indirect cellular mechanisms is discussed. The anti-inflammatory effects of the cholinergic vagal pathways have been demonstrated in the gut; vagotomy and cholinergic antagonist worsen inflammation in animal models of colitis. Furthermore, direct vagal stimulation largely prevents the occurrence of POI via its anti-inflammatory effects. It may be questioned, however, whether these anti-inflammatory effects are based on the anti-inflammatory effect of ACh itself or whether these could be attributed to the release of neuropeptides and neurotransmitters after activation at a postganglionic level. 

Our current understanding of the level of neural regulation of the immune response indicates strong influences of interactive para- and sympathetic innervation. Vagal inhibition of postganglionic catecholaminergic fibers has been recently reported [3, 44]. This concurs with the classic notion of the parasympathetic system opposing or inhibiting the sympathetic system to maintain the physiological homeostasis. However, most recent studies focusing on immunological aspects of neuronal immune modulation indicate that the vagus nerve activates sympathetic splenic nerves to release NE. In septic models, the protective effect of vagal nerve stimulation has been attributed to modulation of splenic release of catecholamines [3]. Hence, the immunomodulatory role of the ENS remains to be fully established, although the ENS is probably involved in the vagal-mediated anti-inflammatory effects. Hence these systems can team together to restrain systemic inflammation, at least in experimental models. 

In conclusion, harnessing the potent immunomodulatory properties of nervous systems as well as endogenous neuropeptides and exploring the use of endogenous resolution mediators offer the best prospects for curbing excessive inflammatory responses, initiating appropriate mucosal wound repair processes, and restoring proper GI homeostasis. However, it is still to be discovered to what extend such “neuroimmunomodulation” is relevant for intestinal disease pathology, and although neuropeptides or their receptors are potential targets of immune modulation, development of drug in that area still awaits further development.

## Figures and Tables

**Table 1:** Summary of immunomodulatory effects of neurotransmitters.

Neurotransmitter	Receptor	Cell source of neurotransmitter	Effects of receptor activation in intestinal disease
Acetylcholine	Nicotinic acetylcholine receptors Muscarinic acetylcholine receptors	Multiple	Vagotomy ameliorates colitis [68] Variable outcomes of nicotinic agonists on the course of colitis [51] Vagal nerve stimulation ameliorates intestinal inflammation: Postoperative ileus, [69] septic ileus Intestinal barrier breakdown [70] Mast cell activation [28] Ischemia reperfusion injury

Dopamine, adrenaline, and noradrenaline	Dopamine receptors, adrenergic receptors a and b	CNS neurons Recruited immune cells Postganglionic Nerves throughout the GI tract	Potent inhibitor of Th1 and macrophage functions Multiple effects on cytokine and chemokine secretion (reviewed a.o. in [30, 32, 71]) In APCs an decrease of IL-12 Type 1, proinflammatory TNF-a Type 1, proinflammatory IFN-g Type 1, proinflammatory IL-2 Type 1 IL-1 Proinflammatory IL-4 Type 2, anti-inflammatory IL-3 Hemopoietic factor GM-Hemopoietic factor CSF MIP-1*α*-Proinflammatory chemokine In APCs an increase of IL-10 Type 2, anti-inflammatory TGF-a Type 2, anti-inflammatory IL-8 Proinflammatory Chemotactic for neutrophils, macrophages, and T lymphocytes [72] Promotes the development of the precursors of granulocytes and macrophages [73]

Vasoactive intestinal peptide	VPAC1 VPAC2	Recruited immune cells Enteric nerve plexus Lamina propria nerves CNS	Amelioration of TNBS-induced colitis by shifting T-cell responses from Th1 to Th2 [74]. Generation of tolerogenic dendritic cells [75]

Substance P	NK-1R NK-2R NK-3R	Recruited immune cells, lamina propria macrophages, colonic glia Motor neurons of intestinal muscularis Lamina propria nerves CNS	Antagonists ameliorate disease in a rat model of TNBS-induced colitis [76] NK-1R^−/−^ mice protected from inflammatory diarrhoea in *C. difficile* toxin A [77] Proinflammatory activation of myeloid cells

Neuropeptide Y Peptide YY Pancreatic polypeptide	6 Y receptors (Y1-6R)	CNS Mononuclear blood leukocytes T cells, APCs	Enhanced T-cell cytokine release Reduced APC cytokine release and function [78, 79]
